# 

**Published:** 1885-02

**Authors:** 


### ILLINOIS STATE DENTAL SOCIETY.

The twenty first annual meeting of the Illinois State Dental Society will be held at Peoria, Ill , commencing Tuesday, May 12th, 1885, and continuing four days.

The State Board of Dental Examiners will be at the National Hotel at 10 A. M. Monday, (May 11th,) at which time candidates for examination must present themselves punctually. The examinations will occupy until Thursday, May 14th.

J. W. WASSALL, *Secret'y,*

103 STATE ST., CHICAGO.

### To Readers and Correspondents.

Communications intended for publication, and exchange journals and papers should be addressed to the Editor of the American Journal of Dental Science, 259 N. Eutaw St., Baltimore, Md. All letters relating to the business of the Journal, or containing cash remittances, to be directed to Messrs. Snowden & Cowman. Articles for publication should be received by the editor at least three weeks previous to the first month on which the number in which it is designed for them to appear, is issued.

☞ The American Journal of Dental Science will contain fifty pages of reading matter in each number, making a volume of about

*Six Hundred* pages,

EXCLUSIVE OF ADVERTISEMENTS.

